# Isoforms of Human MARCH1 Differ in Ability to Restrict Influenza A Viruses Due to Differences in Their N Terminal Cytoplasmic Domain

**DOI:** 10.3390/v14112549

**Published:** 2022-11-18

**Authors:** Fernando Villalón-Letelier, Rubaiyea Farrukee, Sarah L. Londrigan, Andrew G. Brooks, Patrick C. Reading

**Affiliations:** 1 Department of Microbiology and Immunology, The University of Melbourne at the Peter Doherty Institute for Infection and Immunity, 792 Elizabeth St, Melbourne, VIC 3000, Australia; 2 WHO Collaborating Centre for Reference and Research on Influenza, Victorian Infectious Diseases Reference Laboratory, Peter Doherty Institute for Infection and Immunity, 792 Elizabeth St, Melbourne, VIC 3000, Australia

**Keywords:** influenza A virus, E3 ubiquitin ligase, ubiquitination, virus restriction

## Abstract

MARCH1 and MARCH8 are closely related E3 ubiquitin ligases that ubiquitinate an overlapping spectrum of host proteins and restrict replication of certain viruses. While the antiviral activity of MARCH8 has been intensively studied, less is known regarding virus inhibition by MARCH1. Isoforms 1 and 2 of MARCH1 are very similar in overall structure but show major differences in their N-terminal cytoplasmic domain (N-CT). Herein, we used a doxycycline-inducible overexpression system to demonstrate that MARCH1.1 reduces titres of influenza A virus (IAV) released from infected cells whereas MARCH1.2 does not. The deletion of the entire N-CT of MARCH1.2 restored its ability to restrict IAV infectivity and sequential deletions mapped the restoration of IAV inhibition to delete the 16 N-terminal residues within the N-CT of MARCH1.2. While only MARCH1.1 mediated anti-IAV activity, qPCR demonstrated the preferential expression of MARCH1.2 over MARCH1.1 mRNA in unstimulated human peripheral blood mononuclear cells and also in monocyte-derived macrophages. Together, these studies describe the differential ability of MARCH1 isoforms to restrict IAV infectivity for the first time. Moreover, as published immunological, virological and biochemical studies examining the ability of MARCH1 to target particular ligands generally use only one of the two isoforms, these findings have broader implications for our understanding of how MARCH1 isoforms might differ in their ability to modulate particular host and/or viral proteins.

## 1. Introduction

Membrane-associated RING-CH (MARCH) family proteins are RING-finger E3 ubiquitin ligases which share a relatively conserved structure and generally contain a catalytic C4HC3 RING finger (RING-CH motif) domain in their N-terminal cytoplasmic tail [1,2]. To date, 11 MARCH family proteins have been described in humans and it is well-established that these proteins downregulate cell-surface expression of a wide variety of cellular transmembrane proteins. MARCH proteins can be classified into several subgroups, including MARCH1/8, MARCH2/3 and MARCH4/9/11, with MARCH1 and 8 sharing approximately 60% sequence homology [3]. Of these, MARCH1 and MARCH8 have been particularly well studied and show a significant overlap in substrate specificity, including major histocompatibility complex class II (MHCII) molecules, cluster of differentiation (CD) antigens (e.g., CD83, CD86, CD95 and CD98), and other cell-surface molecules, including TRAIL1, the transferrin receptor (TfR) and B-cell receptor-associated protein 31 (Bap31), amongst others (reviewed in [3,4]). More recently, several studies have also demonstrated the ability of MARCH1 and MARCH8 to target the envelope glycoproteins of certain viruses to promote their downregulation from the cell surface, resulting in reduced infectivity of nascent virions released from infected cells. The antiviral activity of MARCH8 has been particularly well studied with human MARCH8 reported to target specific lysine (K) residues in the cytoplasmic tail of some viral envelope glycoproteins, such as vesicular stomatitis virus glycoprotein (VSV-G) [5]. However, human MARCH8 also downregulates human immunodeficiency virus 1 envelope (HIV-1 Env) [5,6,7], Ebola virus glycoprotein (EboV-GP) and severe acute respiratory syndrome coronavirus (SARS-CoV)-2 spike (S) glycoproteins [6], even if the N-terminal cytoplasmic tail (N-CT) of these viral glycoproteins has been deleted. In the case of the influenza A virus (IAV), two studies confirm that human MARCH8 restricts IAV at a late stage in the virus replication cycle, with one study reporting the restriction to be associated with MARCH8-mediated ubiquitination of K78 in the N-CT of the viral M2 protein [8], while the other demonstrated that mutation of this residue alone or in combination with other K residues in the M2 N-CT did not affect MARCH8-mediated restriction of IAV [9].

Compared to MARCH8, less is currently known regarding the ability of other MARCH proteins, including MARCH1, to restrict different viruses. That said, human MARCH1 and MARCH2 inhibit HIV-1 Env and VSV-G pseudotyped viruses by reducing the incorporation of viral envelope glycoproteins into the budding virions [7,10]. Mouse and human MARCH1 and 8, as well as human MARCH2, were also shown to target the envelope glycoproteins on mouse leukemia virus (MLV) whereas human MARCH1, 2 and 8 and mouse MARCH8, but not mouse MARCH1 or 2, were associated with the degradation of HIV-1 gp120 and gp41 [7]. Of note, the C-terminal cytoplasmic tail of the MLV p15 glycoprotein was essential for MARCH-mediated restriction in this study.

While MARCH1 and MARCH8 are generally reported to display overlapping substrate specificity, selective recognition of CD44 and CD81 by MARCH8, but not by MARCH1, has been reported [11,12]. In a recent study, we also reported that inducible overexpression of MARCH8 resulted in the potent inhibition of IAV infectivity whereas the overexpression of MARCH1 did not [9]. Of note, there are two isoforms of MARCH1, namely MARCH1.1 and MARCH1.2, which are very similar in overall structure and homology but show major differences in the sequence of their N-terminal cytoplasmic domain (N-CT). Moreover, studies reporting immunological and biochemical features of MARCH1 in different studies have used only one of the two isoforms or the particular isoform utilised has not been reported. In our previous studies, we only examined the MARCH1.2 isoform for its ability to inhibit IAV [9]. Herein, we confirm that MARCH1.2 contains residues in its N-CT domain that inhibit its ability to restrict IAV replication and its ability to downregulate CD44 and CD81. Moreover, we demonstrate that similar to MARCH8, the MARCH1.1 isoform also mediates potent restriction of IAV infectivity.

## 2. Materials and Methods

**Cells and viruses**

First, 293T cells (ATCC CRL-3216) were maintained and passaged in Dulbecco’s Modified Eagle Medium (DMEM) (Gibco, ThermoFisher Scientific, MA, USA) supplemented with 10% (*v*/*v*) FBS, 2 mM L-glutamine (Gibco) and 1 mM sodium pyruvate (Gibco). For cells engineered to express doxycycline (DOX)-inducible MARCH proteins, Tetracycline System-Approved FBS (Takara Bio USA, CA, USA) was used. Madin–Darby Canine kidney (MDCK) cells (ATCC CCL-34) were maintained and passaged in RPMI 1640 medium supplemented with 10% (*v*/*v*) foetal bovine serum (FBS, Gibco), 2 mM L-glutamine and 1 mM sodium pyruvate. A549 cells were maintained in Ham’s F-12K (Kaighn’s) Medium (Gibco) and THP-1 cells in RPMI 1640 Media (Gibco), both supplemented with 10% (*v*/*v*) FBS, 2 mM L-glutamine (Gibco) and 1 mM sodium pyruvate (Gibco).

**Influenza A viruses (IAV)**

Seasonal IAV strains used in this study were A/Udorn/307/72 (H3N2), A/Beijing/353/89 (H3N2), A/New Caledonia/20/99 (H1N1) and A/Perth/265/2009 (H1N1pdm09). Viruses were obtained from the WHO Collaborating Centre for Reference and Research on Influenza (WHO CCRRI), Melbourne, Australia. Viruses were propagated in the allantoic cavity of 10-day embryonated chicken eggs following standard procedures [13] and titres of infectious virus were determined on MDCK cells by a standard plaque assay and expressed as plaque-forming units (PFU) per mL [14].

**Generation of cell lines with DOX-inducible protein expression.** A three-step cloning strategy was required to generate lentiviruses expressing either MARCH1 isoform 1 (CCDS54814.1), MARCH1 isoform 2 (CCDS3806.1) or MARCH8 (CCDS7213.1). Each MARCH protein contained a N-terminal FLAG tag and was expressed under the control of a tight TRE promoter containing seven tet operator sequences that confer DOX-inducibility as described in [9]. Lentiviral particles were produced by co-transfecting the packaging plasmids pMDL, pRSV-REV and pMD2.G, along with the pFUV1mCherry transfer plasmid using Lipofectamine 2000 (Invitrogen), following the manufacturer’s instructions. At 48 h post-transfection, the cell supernatants harvested were used to transduce 293T cells and mCherry-positive cells were sorted 72 h later using a BD FACSAria III Cell Sorter (BD Biosciences) and were expanded for use. The plasmids were kindly provided by Professor Marco Herold (Walter and Eliza Hall Institute of Medical Research, Melbourne, Australia).

**Detection of DOX-inducible MARCH proteins by flow cytometry.** DOX-inducible 293T cells were seeded in 24-well tissue culture plates (Nunc) and cultured overnight. To induce MARCH protein expression, the cells were incubated in media containing 1 μg/mL of DOX (Sigma-Aldrich, MO, USA) for 24 h at 37 °C and, at 20 h post-DOX induction, 10 μM of the proteasomal inhibitor MG132 (Sigma-Aldrich) was added to reduce intracellular protein degradation. At 24 h post-DOX induction, the cells were detached and stained with fixable viability dye eFlour 780 (eBioscience, CA, USA), washed and fixed with 2% (*v*/*v*) paraformaldehyde in PBS. After permeabilization in 0.5% (*v*/*v*) Triton X-100, the cells were stained with anti-FLAG-Allophycocyanin (APC) mAb (Clone L5, Biolegend, CA, USA), washed and analysed by flow cytometry. The samples were acquired on an LSRFortessa flow cytometer (BD Bioscience) and analysed using FlowJo analysis software version 10.6.2.

**Modulation of cell-surface CD44, CD81 and CD86 expression.** To evaluate the functionality of MARCH proteins after DOX-induction, flow cytometry was used to monitor cell-surface expression of CD44, CD81 and CD86. For CD44 and CD81, DOX-inducible 293T cells were cultured overnight and then incubated in the presence (DOX) or absence (no DOX) of 1 ug/mL of DOX. After 24 h, the cells were detached and stained with fixable viability dye eFlour 780, anti-CD81 conjugated to Brilliant Violet (BV)421 mAb (Clone JSS-81) and anti-CD44 conjugated to BV650 mAb (Clone IM7) and analysed by flow cytometry. For CD86, DOX-inducible 293T cells were transiently transfected with pHAGE-CD86-ZsGreen (kindly provided by Dr. Melissa Call, Walter and Eliza Hall Institute of Medical Research, Melbourne, Australia) which contained an internal ribosome entry site (IRES) allowing for the simultaneous expression of CD86 and the fluorescent protein ZsGreen. At 12 h post-transfection, the cells were incubated in the presence (DOX) or absence (no DOX) of DOX. At 48 h post-transfection, the cells were stained with fixable viability dye eFlour 780 and anti-CD86 conjugated to Brilliant Violet (BV)711 mAb (Clone 2331 (FUN-1), BD Biosciences) and analysed by flow cytometry.

**Virus infection assays.** The cells seeded in 24-well tissue culture plates were washed and then infected with IAV at the indicated multiplicity of infection (MOI, in PFU/cell). After incubation for 1 h at 37 °C, the cells were washed to remove residual inoculum and incubated in serum-free media. In some experiments, media was supplemented with 0.5 μg/mL TPCK trypsin (Worthington Biochemical, NJ, USA) to promote multicycle replication. To evaluate the early stages of virus replication, the percentage of cells expressing newly synthesised IAV nucleoprotein (NP) was measured by flow cytometry at 8 hpi as described [9]. Briefly, virus-infected cells were stained with fixable viability dye eFlour 780 (eBioscience), and then fixed, permeabilized and stained with anti-IAV NP conjugated to FITC (Clone 431, Abcam, UK) before analysis by flow cytometry. To evaluate IAV replication, cell supernatants were collected at indicated times, clarified by centrifugation and titres of infectious virus in cell-free supernatants were determined by a standard plaque assay [15]. Virus titres are expressed as plaque-forming units (PFU) per ml or as VS/mL, respectively.

**Generation of MARCH1.2 mutants with sequential deletions in the N-CT domain.** N-CT deletion mutants of MARCH1.2 were generated by PCR using the Pfu DNA polymerase (Agilent Technologies) according to the manufacturer’s instructions. Each mutant was generated using a reverse primer specific for pcDNA3.1, together with the following forward primers: MARCH1.2-N-CT-Δ15: 5′-AAGGGATCCAAGTCCAAGATCAGCACCATGTACTACC-3′; MARCH1.2-N-CT-Δ16: 5′-AAGGGATCCTCCAAGATCAGCACCATGTACTACC-3′; MARCH1.2-N-CT-Δ29: 5′-AAGGGATCCAAGCTGAGCAACCTGTTTCTGCAAGCC-3′; and MARCH1.2-N-CT-Δ40: 5′-AAGGGATCCCCCACCACCGGCACAGCTCCTAGAAGC-3′. MARCH1.2-Δ-N-CT was generated previously [9]. Cell lines with DOX-inducible expression of mutant versions of MARCH1.2 were generated as described above.

**Detection of endogenous MARCH1.1 and MARCH1.2 mRNA expression by qPCR.** Primers were designed targeting the differential N-CT domain of MARCH1.1 and MARCH1.2. MARCH1.1 and MARCH1.2 open reading frames were also ordered as gene blocks (Thermofisher Scientific, MA, USA) and cloned into pcDNA3.1 (+) hygromycin vector using the Bam HI/EcoRI restriction sites. Plasmid standards with known copy numbers were then used to optimize the qPCR for MARCH1.1 and MARCH1.2 detection. The final primers that were used for subsequent qPCR are as follows MARCH 1.1: Fwd 5′-CGCCTCACAAACCTCCACAT-3′, Rev 5′-TGTTGGGCTGCTTGCTTTTG-3′ and MARCH 1.2: Fwd 5′-ATGACCAGCAGCCACGTTT-3, Rev 5′-CAAGTTAGATAATTTGGCATCTTGG-3′.

SYBR green-based qPCR cycling conditions were as follows: 95 °C for 2 min and then 40 cycles of 95 °C for 5 s and 65 °C for 20 s. Data acquisition was performed using the QuantStudio 7 Pro Real-Time PCR System (Applied Biosystems, CA, USA). The CT values from the plasmid standards were plotted against the known copy numbers to generate a standard curve, and the slope of the curve was used to calculate PCR efficiency as follows: E = −1 + 10^(−1/slope)^.

Optimised primers and qPCR conditions were then used to measure expression levels of endogenous MARCH1.1 and MARCH1.2 mRNA in the following cell lines: A549 cells, THP-1 non differentiated cells, PMA-differentiated THP-1 cells and peripheral blood mononuclear cells (PBMCs) from different donors. PBMCs were isolated from healthy blood donors (with approval from the School of Biomedical Sciences Human Ethics Advisory Group, The University of Melbourne, Australia) using Ficoll-Paque density gradient centrifugation and differentiated THP-1 cells were obtained by seeding 3.5 × 10^5^ cells into 24-well tissue culture plates and incubating overnight with 25 ng/mL PMA. RNA was extracted from these cells using the RNeasy Plus Mini Kit (Qiagen, Germany), treated with amplification grade DNase I (Sigma Aldrich, MO, USA) and RNA concentration was then standardised across samples. The RNA was then converted to cDNA using the SensiFAST cDNA Synthesis Kit (Bioline, TN, USA). The SYBR-based qPCR was run on the standardized samples as described above. For each run, the standards for both isoforms were included so absolute RNA copy numbers could be calculated from standard curves.

To test for the induction of MARCH1 proteins, human-monocyte-derived macrophages were derived from PBMCs by positive selection of CD14^+^ monocytes using CD14 microbeads (Miltenyi Biotec, Germany). The CD14^+^ monocytes were cultured in RPMI supplemented with 10% human serum (Sigma) for 6–7 days. The hMDM were then either treated with 1000 U/mL IFN-α (Lonza, Switzerland), infected with IAV (Beij89, MOI 10 PFU/cell), or incubated with media (mock). After 6 or 24 h, the total RNA was extracted and qPCR was performed as described above.

**Statistical analysis.** Graphs and statistical analysis (as indicated in the figure legends) were performed using either GraphPad Prism (GraphPad software) version 9.0.2 or Rstudio Version 1.3.1093.

## 3. Results

**Characterisation of cell lines with inducible expression of different MARCH1 isoforms.**

**Figure 1:**
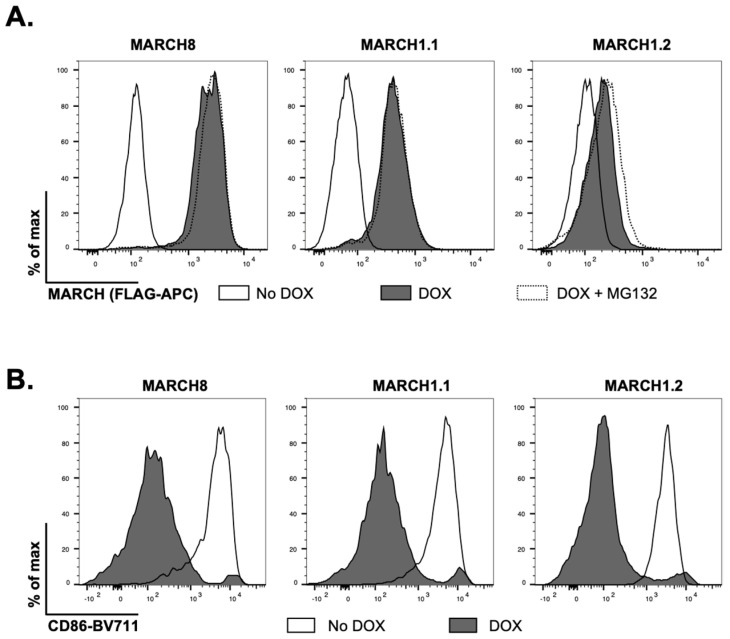
**Characterisation of cell lines with inducible expression of MARCH1.1 or MARCH1.2.** (**A**) 293T cells with stable DOX-inducible overexpression of FLAG-tagged MARCH1.1, 1.2 or 8 proteins were cultured for 24 h in media alone (no DOX) or supplemented with 1 mg/mL DOX (DOX) or in DOX with MG132 added for the last 4 h (DOX + MG132). MARCH protein expression was assessed by intracellular staining with anti-FLAG antibody in conjunction with flow cytometry. (**B**) The cells were transfected with a plasmid encoding CD86 and ZsGreen fluorescent protein. At 12 h post-transfection, MARCH expression was induced with DOX as above and 24 h later cells were stained for cell surface CD86. For (**A**,**B**), representative histograms from >2 independent experiments are shown.

We generated 293T cell lines with stable doxycycline (DOX)-inducible overexpression of MARCH1.1 and MARCH1.2, along with MARCH8 as a control host protein known to restrict the release of infectious IAV from virus-infected cells [9]. As each protein was engineered to express a N-terminal FLAG-tag, we first used flow cytometry to confirm DOX-inducible overexpression in each instance. MARCH8 and MARCH1.1 were expressed at high levels and the addition of MG132 to the media 4 h prior to analysis (to inhibit proteosomal degradation) did not enhance the levels of either protein (Figure 1A). In contrast, MARCH1.2 was expressed at lower levels and culture in MG132 did enhance intracellular protein expression (Figure 1A). Moreover, the induced MARCH1.1 and 1.2 proteins, as well as MARCH8 protein, all downregulated cell-surface expression of CD86, confirming the functionality of each individual MARCH protein (Figure 1B).

**Isoforms of MARCH1 differ in their ability to restrict the late stages of IAV replication in host cells.**

**Figure 2:**
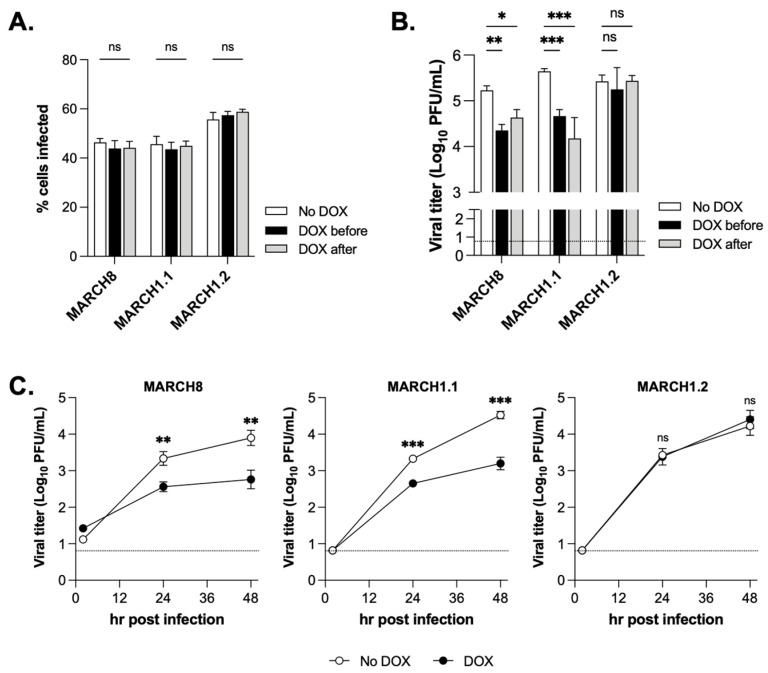
**Isoforms of MARCH1 differ in their ability to restrict the late stages of IAV replication in host cells**. (**A**,**B**) Cells cultured in media alone (no DOX, white bars) or DOX-induced for 24 h (DOX before, black bars) were infected with IAV strain Beij89 at (**A**) MOI = 1, or (**B**) MOI = 5 PFU/cell for 1 h at 37 °C, then washed and cultured at 37 °C. In some cells cultured and infected in media alone, DOX was added immediately after washing away virus inoculum (DOX after, grey bars). (**A**) At 8 hpi, the cells were washed, fixed, permeabilized and stained with FITC-labelled mAbs to viral NP and the percentage of virus infected cells was determined by flow cytometry. Data show the mean ± SD from triplicate samples. (**B**) At 24 hpi, the supernatants were harvested, clarified and titres of infectious virus determined by plaque assay on MDCK cells. Data show the mean ± SD from triplicate samples. For (**A**,**B**), the results show two-way ANOVA and Bonferroni’s post-test, no DOX to DOX. (**C**) Cells cultured in media (no DOX, white circles) or DOX-induced for 24 h (DOX, black circles) were infected with Beij89 (MOI = 0.01) and cultured in the presence of exogenous trypsin (0.5 mg/mL) to enable multicycle replication. Virus titres in clarified supernatants from triplicate samples harvested at 2, 24 and 48 hpi were determined by plaque assay. Dashed lines in (**B**,**C**) show the detection limit of the plaque assay. Two-tailed unpaired Student’s *t*-test for no DOX vs. DOX at each timepoint. All data are representative of two or more independent experiments. * = *p* < 0.05, ** = *p* < 0.01. *** = *p* < 0.001, ns = not significant.

Next, the cells were (i) pre-treated with DOX for 24 h and then infected with IAV strain A/Beijing/353/1989 (Beij/89; DOX before), or (ii) cultured overnight in DOX-free media, infected with Beij/89 and then cultured in DOX (DOX after), or (iii) not induced with DOX at any time (no DOX) and the percentage of IAV-infected cells was determined by flow cytometry at 8 h post-infection (hpi). DOX-inducible expression of MARCH1.1, MARCH1.2 or MARCH8 did not impact the percentage of virus-infected cells detected at 8 hpi (Figure 2A) confirming that these proteins did not impact virus replication at the steps prior to the synthesis of viral nucleoprotein (NP) in the infected cells. To investigate whether MARCH1 isoforms differed in their ability to restrict IAV infectivity, virus titres were determined at 2 versus 24 hpi following the infection of uninduced (no DOX) 293T cells expressing MARCH1.1, MARCH1.2 or MARCH8, or the same cells induced with DOX 24 h prior to (DOX before) or immediately after infection (DOX after). For all cell lines, virus titres increased markedly between 2 and 24 hpi, consistent with productive virus replication (data not shown). As previously reported [9], inducible MARCH8 resulted in a significant reduction in the titres of infectious IAV released at 24 hpi, whereas MARCH1.2 did not (Figure 2B). However, DOX-inducible overexpression of MARCH1.1 also resulted in a significant reduction in titres released from IAV-infected cells. These findings were confirmed when the cells were infected with a low MOI (0.1 PFU/cell) in the presence of exogenous trypsin to promote multicycle virus replication between 2 and 48 hpi (Figure 2C). Together, these results confirm that MARCH1 isoforms differ in their ability to restrict IAV and that only MARCH1.1 can effectively inhibit a late stage of the IAV replication cycle.

**Differences in the N-terminal cytoplasmic domain (N-CT) between MARCH1.1 and MARCH1.2 are the key determinants of their differential ability to modulate CD44 and CD81, and to mediate IAV restriction**

**Figure 3:**
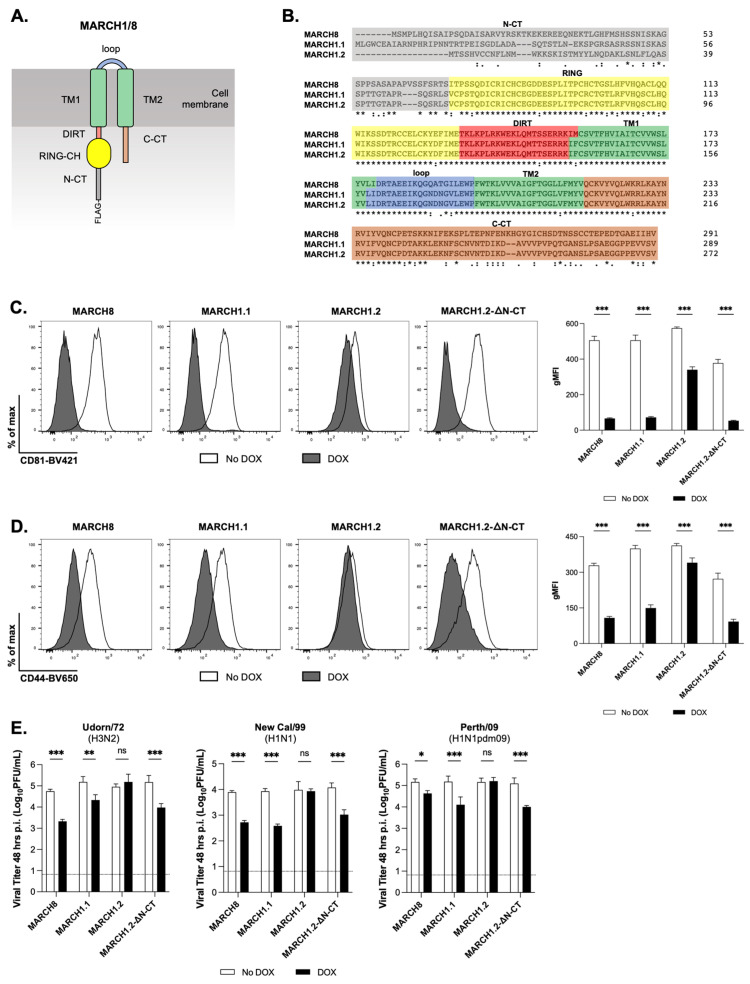
**Differences in the N-terminal cytoplasmic domain (N-CT) between MARCH1.1 and MARCH1.2 are the key determinants of their differential ability to modulate CD44 and CD81, and to restrict IAV growth**. (**A**) Schematic of the predicted topologies of MARCH1/8 highlighting domain structure and location of N-terminal FLAG tag used in our studies. (**B**) Sequence alignment of human MARCH8, MARCH1.1 and MARCH1.2. proteins using CLUSTAL OMEGA 1.2.4. In (**A**,**B**), N-terminal cytoplasmic tail (N-CT) is grey; RING-CH domain is yellow; the domain in between the RING-CH and the first transmembrane domain (DIRT) is red; Transmembrane domains (TM1/2) are green, the extraluminal connecting loop is blue and C-terminal cytoplasmic tail (C-CT) is brown. (**C**,**D**) Here, 293T cells cultured in media alone (no DOX) or supplemented with 1 mg/mL DOX (DOX) for 24 h were detached, washed and stained for cell-surface expression of CD81 (**C**), or CD44 (**D**). Representative histograms and geometric mean fluoresce intensity (gMFI ± SD) from triplicate samples are shown. (**E**) Cells cultured in media (no DOX, white bars) or DOX-induced for 24 h (DOX, black bars) were infected with Udorn/72 (H3N2), New Cal/99 (H1N1) or Perth/09 (H1N1pdm09) (MOI = 0.01), washed and then virus titres in clarified supernatants from triplicate samples harvested at 24 hpi were determined by plaque assay. The results show two-way ANOVA and Bonferroni’s post-test, no DOX to DOX. * = *p* < 0.05, ** = *p* < 0.01. *** = *p* < 0.001, ns = not significant. All data representative of two or more independent experiments.

MARCH1 and MARCH8 share a common domain structure comprising a N-terminal cytoplasmic tail (N-CT), followed by the RING-CH domain, the domain in between the RING-CH and the first TM domain (DIRT), two transmembrane domains (TM1/2), an extraluminal connecting loop between the TM domains (loop) and a C-terminal cytoplasmic tail (C-CT) (Figure 3A). Moreover, the two isoforms of MARCH1 show a high degree of homology across the RING-CH, DIRT, TM1/TM2, loop and C-CT domains (100% identity at amino acid level) but show only 37.7% identity across their N-CT domain (Figure 3B). While MARCH1 and 8 generally show overlapping substrate specificity, it has been reported that MARCH8 selectively targets CD44 and CD81 [11,12]. Given the differences observed between MARCH1 isoforms in their ability to restrict IAV infectivity (Figure 2), we next compared the cells with an inducible expression of MARCH1.1 and 1.2, as well as MARCH8, for their ability to modulate cell-surface CD44 and CD81. In these experiments, we also assessed the cells expressing a deletion mutant of MARCH1.2 lacking 56 residues from its N-CT domain (MARCH1.2-DN-CT). While inducible MARCH1.2 induced a very modest (albeit significant) downregulation of CD44 or CD81, both MARCH1.1 and MARCH8 expression resulted in potent downregulation of either protein from the cell-surface (Figure 3C, left and right panels, respectively). Moreover, the MARCH1.2-DN-CT also mediated potent downregulation of both CD44 and CD81 (Figure 3D, middle panels). These data demonstrate that similar to MARCH8, the expression of MARCH1.1 results in the potent downregulation of both CD44 and CD81 from the cell surface. In addition, we confirm that inducible MARCH1.2 does not downregulate CD44 or CD81 and that the deletion of the MARCH1.2 N-CT domain restored its ability to target both CD44 and CD81.

We next assessed the replication of different IAV strains in cells expressing MARCH1.2-DN-CT. Consistent with results using Beij/89 (H3N2, Figure 2B), inducible MARCH8 and MARCH1.1 inhibited IAV strains Udorn/72 (H3N2), New Cal/1999 (H1N1) and Perth/09 (H1N1pdm09), whereas MARCH1.2 did not (Figure 3E). However, the infection of cells expressing inducible MARCH1.2-DN-CT resulted in the inhibition of all IAV strains tested, indicating that the N-CT domain of MARCH1.2 inhibits its ability to restrict replication of different IAV subtypes associated with seasonal influenza in the human population.

**Deletion of 16 residues from the N-terminal of the MARCH1.2 N-CT domain restores its ability to restrict IAV replication**

**Figure 4:**
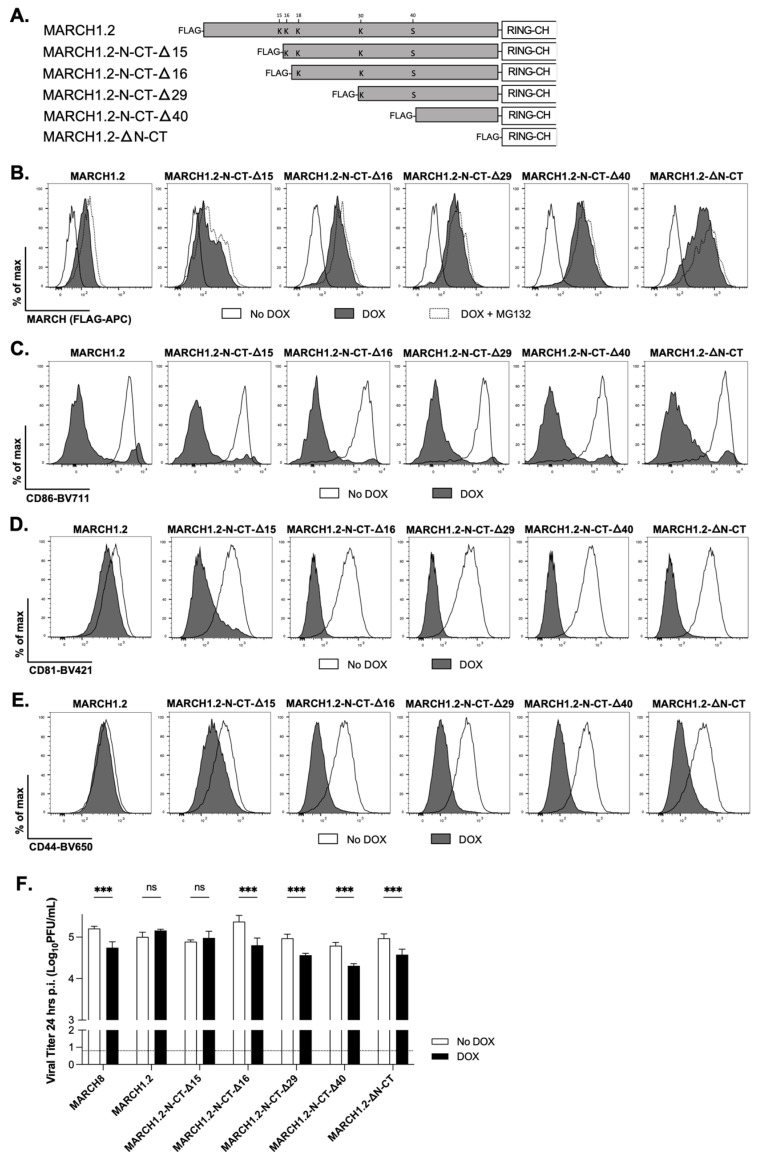
**Deletion of 16 residues from the N-terminal of the MARCH1.2 N-CT domain restores its ability to restrict IAV replication**. (**A**) Schematic of the different MARCH1.2 deletion mutants utilised in this study. (**B**) Stable 293T cells with DOX-inducible expression of MARCH1.2 WT or different MARCH1.2 N-CT deletion mutants were cultured for 24 h in media alone (no DOX), supplemented with 1 mg/mL DOX (DOX) or with DOX in the presence of MG132 for the last 4 h of DOX induction (DOX + MG132). Protein expression of WT and MARCH1.2 mutants was determined by intracellular staining with an anti-FLAG mAb followed by flow cytometry. (**C**) The cells were transfected with a plasmid encoding CD86 and ZsGreen fluorescent protein. At 12 h post-transfection, MARCH expression was (DOX) or was not (no DOX) induced as above and 24 h later the cells were stained for cell-surface CD86 expression. (**D**,**E**) No DOX or DOX-induced cells were detached, washed and stained for cell-surface expression of CD81 (**D**), or CD44 (**E**). In (**C**–**E**) representative histograms from triplicate samples are shown. (**F**) Cells cultured overnight were infected with IAV strain Beij/89 (MOI = 5 PFU/cell) for 1 h at 37 °C, washed and then cultured in media alone (no DOX, white bars) or media supplemented with 1 mg/mL DOX (DOX, black bars). At 24 hpi, the cell supernatants were harvested, clarified by centrifugation and viral titres were determined by plaque assay. The results show two-way ANOVA and Bonferroni’s post-test, no DOX to DOX. *** = *p* < 0.001, ns = not significant. All data are representative of two or more independent experiments.

Given that the deletion of the entire MARCH1.2 N-CT resulted in potent IAV restriction, we aimed to identify the minimal determinant within the N-CT required for its anti-IAV activity. To do this, we generated constructs with stepwise deletions in MARCH1.2 corresponding to the loss of 15, 16, 29 and 40 residues from the N-CT of MARCH1.2 (Figure 4A). Given that autoubiquitination of MARCH1 has been associated with regulating its own intracellular expression levels [16], we sequentially removed specific lysine (K) residues (which might serve as potential targets for autoubiquitination) from the N-CT and then generated 293T cell lines with inducible expression of MARCH1.2 N-CT-D15, N-CT-D16, N-CT D29 and N-CT-D40, respectively. First, we assessed cell lines expressing N-CT deletion mutants of MARCH1.2 for FLAG expression, including wild-type MARCH1.2 and MARCH8, as well as MARCH1.2 DN-CT which lacks the entire 54 residues of the N-CT domain. Compared to wild-type MARCH1.2, the cells expressing MARCH1.2 N-CT-D15 showed evidence of increased FLAG staining and this was markedly enhanced in cells expressing N-CT-D16, N-CT-D29, N-CT-D40 and DN-CT (Figure 4B). The addition of MG132 to media to limit proteosomal degradation resulted in the modest enhancement of MARCH1.2 and N-CT-D15 expression with negligible effects on the other deletion mutants tested. Moreover, all DOX-inducible MARCH proteins downregulated cell-surface CD86 (Figure 4C), confirming protein functionality despite any differences in the levels of protein expression. When examining other ligands, all MARCH1 deletion mutants downregulated cell-surface CD81 (Figure 4D), whereas N-CT-D15 induced only modest downregulation of CD44 (Figure 4E), while all other deletion mutants downregulated CD44 effectively. Thus, while all MARCH1.2 mutants modulate CD86 and CD81 expression, the ability of N-CT-D15 to downregulate CD44 was reduced compared to other mutants.

Next, cells expressing wild-type MARCH1.2 or MARCH8, or different MARCH1.2 N-CT deletion mutants, were infected with Beij/89 and then DOX added immediately after infection to induce MARCH protein expression. As seen in Figure 4F, the expression of wild-type MARCH8, but not MARCH1.2, was associated with reduced IAV titres at 24 hpi. While MARCH1.2 deletion mutants lacking the entire N-CT domain, or lacking the 40, 29 or 16 N-terminal residues of the MARCH1.2 N-CT, all restricted IAV replication, the MARCH1.2 N-CT-D15 mutant did not. These data indicate that the deletion of the first 16 N-terminal residues of the MARCH1.2 N-CT domain is the minimum requirement to restore its ability to restrict IAV replication.

**Figure 5:**
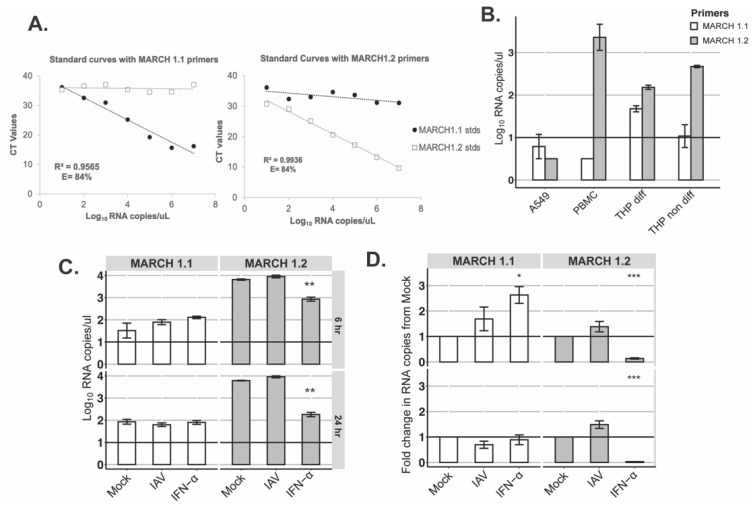
**Endogenous expression MARCH1 isoforms in different human cell lines and primary cells following induction was measured by qPCR**. (**A**) Primers specific to MARCH1.1 (left panel) or MARCH1.2 (right panel) isoforms were designed and qPCR efficiency and specificity was tested using plasmid standards encoding MARCH1.1 or MARCH1.2. Data confirm primer specificity and PCR efficiency was calculated as 84% and 87% for MARCH 1.1 and MARCH1.2 primers, respectively. Data are representative of >two independent experiments (**B**) Endogenous expression levels of MARCH1 isoforms in different cell lines were measured using the optimised qPCR primers and plasmid standards to determine absolute RNA copy numbers. For qPCR, the total RNA was extracted from cells, and 50 ng/µL of each sample was DNase-treated and then used for cDNA synthesis. Data show the mean ± SD from triplicate samples for cell lines (A549, THP-1), from four independent donors (PBMC). (**C**,**D**) The induction of MARCH1 isoforms following IAV infection or IFN-a treatment as determined by qPCR. Monocyte-derived macrophages (MDMs) were cultured in 24-well plates and infected with IAV (Beijing/89, MOI 10) or treated with 1000 U/mL IFN-a for 6 or 24 h. After incubation, the total RNA was extracted and 50 ng/µL of each sample was DNase-treated and then used for cDNA synthesis, prior to qPCR. Data show the mean ± SD of triplicate samples from one donor as (**C**) absolute RNA copy numbers, or (**D**) fold-change from mock. The results are shown for two-way ANOVA and Bonferroni’s post-test, no DOX to DOX. * = *p* < 0.05, ** = *p* < 0.01. *** = *p* < 0.001.

**MARCH1.2 shows preferential expression over MARCH1.1 in human peripheral blood mononuclear cells and macrophages.** MARCH1.1 and MARCH1.2 proteins expressed in 293T cells following DOX induction represent functional proteins (as determined by downregulation of cell-surface CD86), however only MARCH1.1 inhibited the release of IAV from virus-infected cells. Moreover, MARCH1.1 and MARCH1.2 differ markedly in the sequence of their N-CT domain and removal of the last 16 N-terminal residues from the N-CT domain of MARCH1.2 restored its ability to restrict IAV replication. To examine the levels of endogenous MARCH1, we designed primers to unique regions in the N-CT of each isoform and used plasmids expressing either MARCH1.1 or MARCH1.2 to generate a standard curve and to confirm the specificity of each reaction for one of the two MARCH1 isoforms (Figure 5A). Next, we assessed expression of each isoform in A549 cells, undifferentiated and PMA-differentiated THP-1 cells and in peripheral blood mononuclear cells (PBMC) isolated from four independent donors, noting that the results are expressed as log_10_RNA copies rather than normalised to a housekeeping gene. As seen in Figure 5B, MARCH1.1 mRNA was expressed at relatively low levels in all cell types examined, with the highest levels observed in differentiated THP-1 cells. MARCH1.2 expression was markedly higher than MARCH1.1 in THP-1 cells and in PBMC, confirming it to be the major isoform expressed at the mRNA level in these cells (Figure 5B). Given that previous studies reported the induction of MARCH1 in monocyte-derived macrophages (MDM) following overnight exposure to IFN-a [10], MDM were also (i) treated with 1000 U/mL of human IFN-a, or (ii) infected with IAV (Beij/89, MOI = 10), and levels of each MARCH1 isoform were determined 6 and 24 h later. When examining the RNA copy number (Figure 5C), MARCH1.1 showed a trend for increased expression 6 h, but not 24 h, after exposure to IAV or IFN-a and this was significant for IFN-a treatment at 6 h, but not 24 h, when comparing the fold-change relative to mock (Figure 5D). MARCH1.2 expression also showed a trend for increased expression in response to IAV, but was significantly reduced in response to IFN-a when assessed by RNA copy number (Figure 5C) or fold-change relative to mock (Figure 5D) at 6 or 24 h.

## 4. Discussion

MARCH1 and MARCH8 are closely related across their RING-CH and TM domains (>90% sequence identity), but their N-CT and C-CT regions are <20% identical [17]. Moreover, MARCH1 and 8 show a high degree of substrate overlap and share recognition of diverse immunological ligands including Class II MHC, CD83, CD86 (B7.2), CD95, CD98, TfR, TRAIL R1 and Bap31 (reviewed in [4]). However, there is evidence to support differential recognition of particular targets. For example, a large-scale RNA interference screen identified MARCH1 and MARCH9, but not MARCH8, as negative regulators of the insulin receptor (INSR) [18]. To date, only MARCH8 has been reported to mediate recognition of interleukin 1 receptor accessory protein (IL1RAP) [19,20] and E-cadherin [21], although it appears that the activity of MARCH1 was not examined in these studies. Of interest, Bartee et al. reported that MARCH1 and 8, as well as several other MARCH proteins, recognized Bap31, whereas the transfection of cells with MARCH8, but not MARCH1, resulted in the downregulation of CD44 and CD81 [11]. Given this result was consistent with our findings that MARCH8, but not MARCH1, inhibited IAV infectivity [9], we conducted further investigations as to why MARCH1 might not recognise particular ligands known to be targeted by MARCH8.

Two isoforms of MARCH1 have been described however studies to date have not formally addressed the similarities or differences in their biological activities. In fact, the immunological, virological and biochemical studies which have examined the ability of MARCH1 to target particular ligands have used only one of the two isoforms or the particular isoform utilised has not been reported. Of note, a number of studies examining different aspects of MARCH1 biology [10,11,12,17,22] have utilised only the MARCH1.2 isoform. In these studies, both MARCH8 and MARCH1.2 were reported to target Bap31 [11], Tfr, CD86 (B7.2), CD95 (Fas) [17] and CD98 [12], however only MARCH8 expression was associated with the downregulation of cell-surface CD44 [11,12] and CD81 [11]. Herein, our comparison of inducible MARCH1.1 and 1.2 confirms that both downregulated cell-surface CD86, however only MARCH1.1 was also associated with potent downregulation of CD44 and CD81. Clearly the MARCH1.2 isoform encodes a functional protein that recognises a broad range of immunological targets however specific interactions with particular proteins, including CD44 and CD81, are inhibited by residues within its N-CT domain. 

MARCH8 has been reported to downregulate envelope glycoproteins from a broad range of viruses, including HIV-1 Env [23], EboV-GP and SARS-CoV-2 S [6], amongst others. Currently, less is known regarding the antiviral activity of MARCH1, although emerging evidence indicates it can inhibit certain viruses in a similar manner to that reported for MARCH8. For example, Umthong et al. utilised co-transfection studies to demonstrate that human MARCH1.1, along with MARCH2 and 8, restricted the expression of HIV-1 gp120 and gp41, as well as the expression of EboV GP, Nipah virus fusion (F) and attachment (G) glycoproteins, and the IAV HA glycoprotein, amongst others [7]. Following on from studies confirming that MARCH1.2 downregulated Tfr expression [22], it was also shown to downregulate cell-surface HIV-1 Env, thereby inhibiting its incorporation into newly formed virions and resulting in decreased viral infectivity [10], exactly as reported for MARCH8 [23]. Clearly the mechanisms governing the downregulation of HIV-1 Env by MARCH1 are distinct to those involved in the inhibition of IAV as both MARCH1 isoforms appear to restrict HIV-1. Moreover, these findings suggest that residues in the N-CT of MARCH1.2 inhibit its ability to restrict IAV, but not to restrict HIV-1.

The deletion of the entire MARCH1.2 N-CT resulted in the effective downregulation of CD44 (Figure 3C) and CD81 (Figure 3D), as well as the recovery of IAV restriction (Figure 3E). When investigating N-CT residues involved in inhibiting particular functions of MARCH1.2, we report that the removal of the first 15 residues (up to K15) of the MARCH1.2 N-CT was sufficient to restore effective downregulation of CD81 (Figure 4D), while removal of the first 16 residues (up to K16) was required to restore the downregulation of CD44 (Figure 4E) as well as the restriction of IAV growth (Figure 4F). With this background, it is interesting to note that the levels of intracellular MARCH protein (determined by FLAG staining) increased markedly following the deletion of the entire N-CT, a finding similar to that reported previously for mouse MARCH1 [24]. Moreover, FLAG expression increased progressively in cells expressing wild-type, N-CT-D15 and N-CT-D16 MARCH1.2, but not in mutants with additional N-CT residues deleted (Figure 3A), indicating that the first 16 residues contain sequence elements that determine protein stability and/or turnover. While our studies are unable to effectively uncouple the effects of K15/K16 on intracellular MARCH protein expression levels versus the ability of different mutants to mediate biological functions, it should be noted that all N-CT mutants were effective in mediating the downregulation of cell-surface CD86 (Figure 3B), indicating sufficient expression levels of wild-type MARCH1.2 protein for at least some of its biological functions. Given that previous studies demonstrated that MARCH1 regulates its own expression via dimerization and autoubiquitination [16], our findings would suggest that K15 and K16 may be critical sites for autoubiquitination and therefore the regulation of human MARCH1.2 expression.

We propose that the first 16 residues of the N-CT of MARCH1.2 contain residues that inhibit (i) intracellular protein expression levels, and (ii) IAV restriction. The specific residues in the MARCH1.2 N-CT that determine these two characteristics may or may not be the same. As discussed above, given that the expression of mouse MARCH1 is modulated by autoubiquitination [24], we propose that this might also occur for human MARCH1.2 via K15/K16 residues in its N-CT. For IAV restriction, it is possible that the reduced protein levels of MARCH1.2 and/or an additional inhibitory activity present within the first 16 residues of the MARCH1.2 N-CT inhibit its ability to effectively inhibit IAV growth. Similar to other studies examining the functionality of MARCH1 [24,25,26] and MARCH8 [22,27], we generated MARCH1.2 deletion mutants to gain insight regarding residues which impact the regulation of CD44/CD81 expression, as well as IAV restriction. In future studies, the generation of lysine (K)-to-arginine (R) substitution mutants represents an additional approach that may be insightful regarding whether the inhibitory activity maps to specific K residues or to other residues in the N-CT of MARCH1.2.

Given the differential antiviral activity of MARCH1.1 and 1.2 against IAV, we designed RT-PCR assays targeting the N-CT region to discriminate between each isoform and assess mRNA levels in human cells. First, MARCH1.2 was expressed in PBMC from different donors and in MDM at higher levels than the canonical MARCH1.1 (Figure 5B). Moreover, IFN-a treatment inhibited MARCH1.2, but did not modulate MARCH1.1 mRNA levels. Thus, while we have demonstrated that the MARCH1.1 isoform is the only one to restrict IAV growth, endogenous MARCH1.1 is expressed at lower levels within the different human cells tested in our studies, at least at the mRNA level. Protein levels are, however, the key determinant of biological functions and enhanced MARCH1.2 mRNA transcripts may be required to maintain sufficient MARCH1.2 protein levels for biological functions in the face of autoubiquitination and rapid intracellular turnover compared to MARCH1.1. Our studies examining the expression of FLAG-tagged inducible proteins certainly indicate that MARCH1.2 is expressed at low levels compared to MARCH1.1, and that removal of the entire N-CT (or just the first 16 residues up to K16) (Figure 4B) is required for enhanced intracellular protein expression. In future studies it will be important to investigate the relative expression levels and functionality of endogenous MARCH1.1 versus MARCH1.2 proteins in particular cell types, and to determine if they show other differences in their spectrum of target specificity. Of note, discriminating between MARCH1 isoforms at the protein level using specific antibodies is complicated by their high level of amino acid sequence identity, as the two proteins differ only in the sequence of their N-CT domain (Figure 3B).

Overall, our studies clearly demonstrate the differential ability of MARCH1 isoforms to modulate some cellular proteins (i.e., CD44 and CD81) and to restrict IAV infectivity for the first time. In future studies, it will be of interest to assess virus replication, as well as levels of cellular and soluble inflammatory mediators in the airways, in response to the intranasal infection of MARCH1^-/-^ mice with IAV. Differential regulation of CD44 and/or CD81 should also be assessed in vivo using MARCH1^-/-^ mice as this might have important implications for other diseases, including inflammatory conditions. Moreover, as published immunological, virological and biochemical studies examining the ability of MARCH1 to target particular ligands generally use only one of the two isoforms, these findings have broader implications for our understanding of how MARCH1 isoforms might differ in their ability to modulate particular host and/or viral proteins.

## Data Availability

Not applicable.
